# Supplementary figures and images for: Insights from a comprehensive study of Trypanosoma cruzi: A new mitochondrial clade restricted to North and Central America and genetic structure of TcI in the region
Source: PLoS Negl Trop Dis. 2021 Dec 17;15(12):e0010043. doi: 10.1371/journal.pntd.0010043 (PMC8719664; doi:10.1371/journal.pntd.0010043)

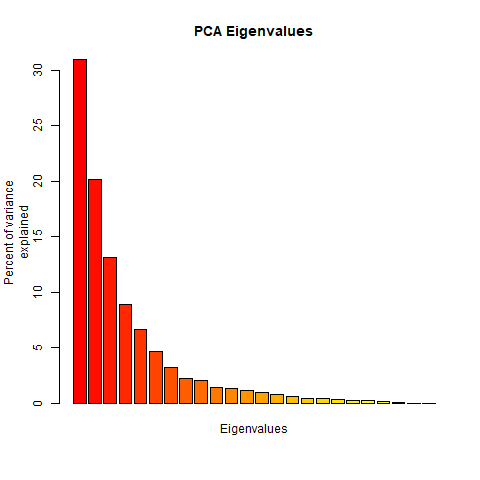

Supplement: S1 Fig — (TIF) [file pntd.0010043.s001.tif]

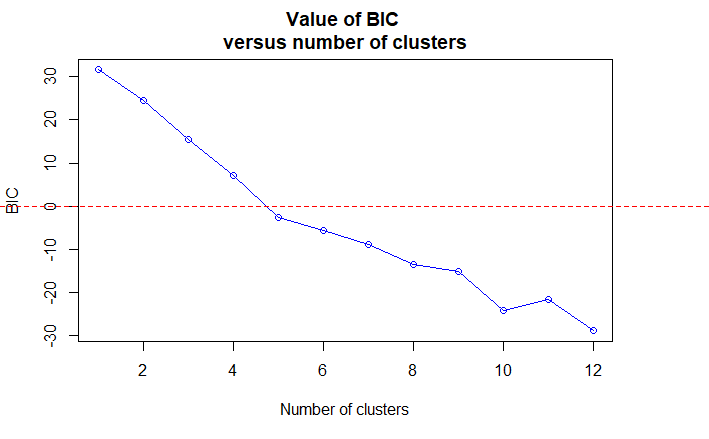

Supplement: S2 Fig — (TIFF) [file pntd.0010043.s002.tiff]

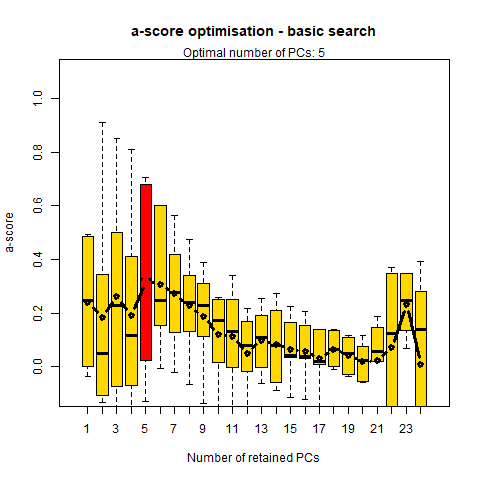

Supplement: S3 Fig — (TIF) [file pntd.0010043.s003.tif]

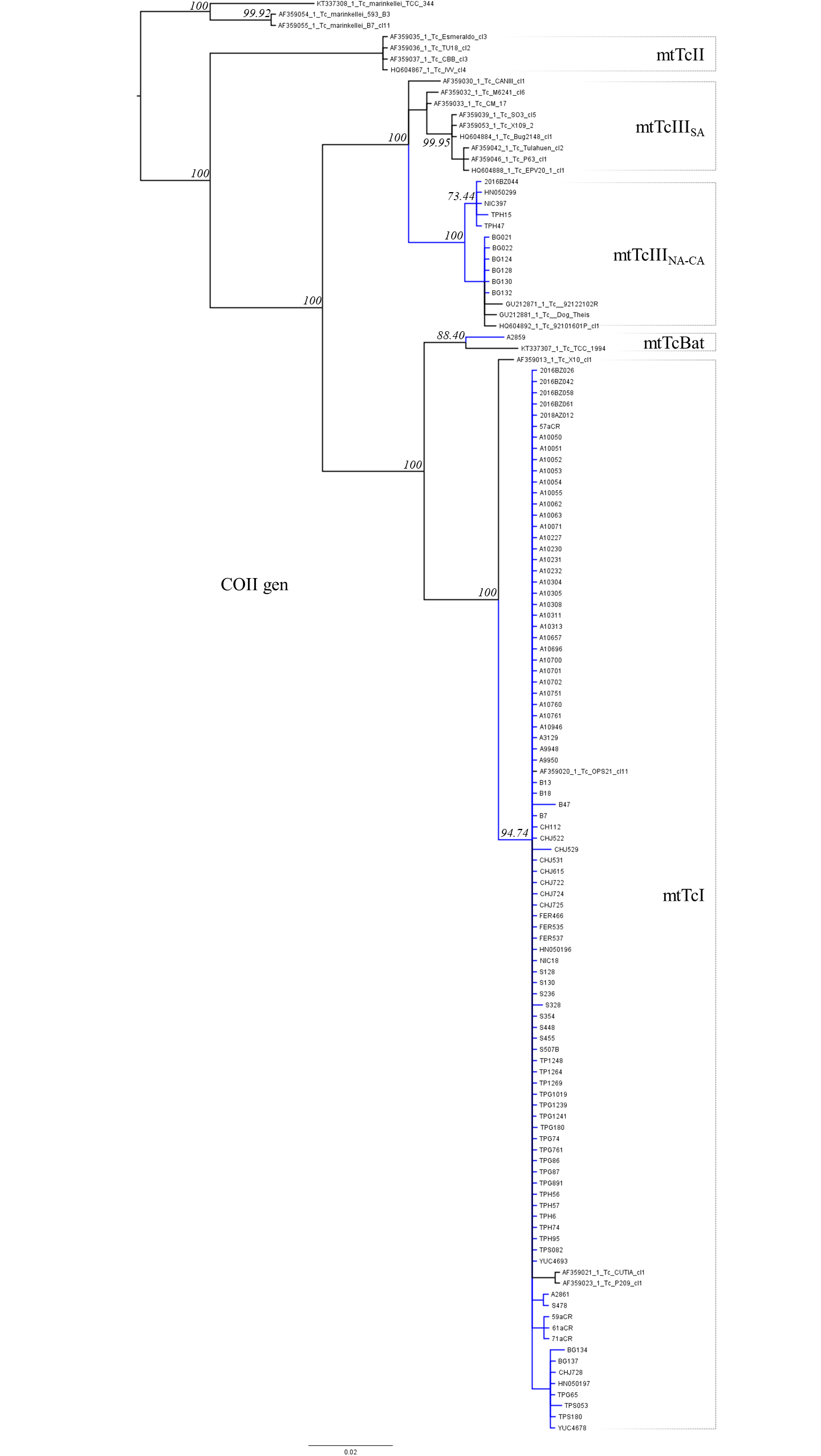

Supplement: S4 Fig — Mitochondrial phylogeny based on COII gene, inferred under the GTR model from 513 nucleotides from reference and newly sequenced samples. (TIF) [file pntd.0010043.s004.tif]

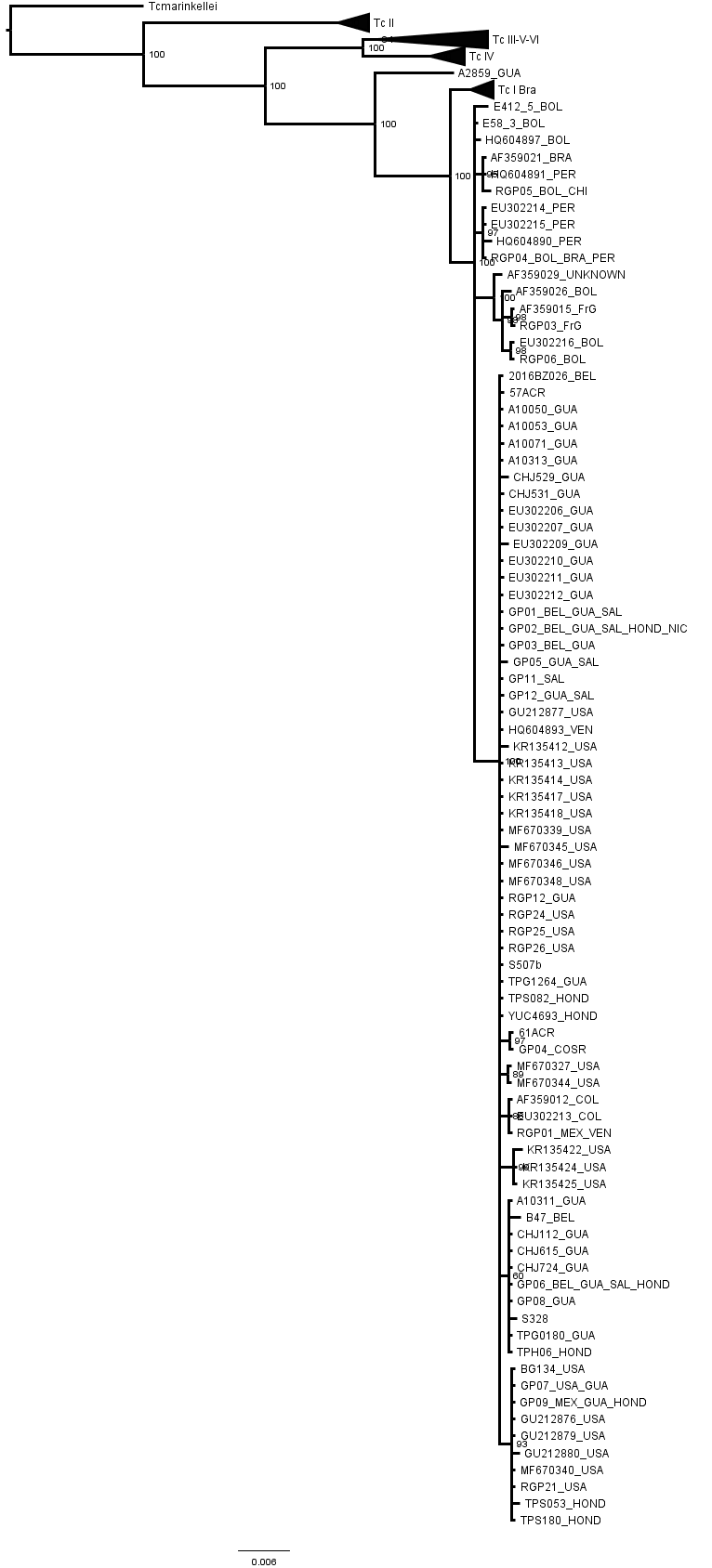

Supplement: S5 Fig — Mitochondrial Phylogeny based on COII-ND1 genes, inferred under the HKY model from 866 nucleotides from over 210 samples total (reference and newly sequenced samples). (TIF) [file pntd.0010043.s005.tif]
