# Supplementary material for: Insights from a comprehensive study of Trypanosoma cruzi: A new mitochondrial clade restricted to North and Central America and genetic structure of TcI in the region
Source: PLoS Negl Trop Dis. 2021 Dec 17;15(12):e0010043. doi: 10.1371/journal.pntd.0010043 (PMC8719664; doi:10.1371/journal.pntd.0010043)
Supplement: Attachment — Submitted filename: Answers_to_reviewers.docx [file pntd.0010043.s009.docx]

October 2021

Dear Dr. Dutra and Dr. Rodriguez,

Deputy Editor
PLOS Neglected Tropical Diseases

We appreciated the helpful comments from you on the previous draft of our manuscript PNTD-D-21-01053 "Insights from a comprehensive study of *Trypanosoma cruzi*: a new mitochondrial clade restricted to North and Central America and genetic structure of TcI in the region". We have addressed these comments in this revised version of the manuscript.

Below please find our responses to the specific comments indicating how we addressed each one. Comments are in *italics* and our responses are marked with >>.

The work is original and has not been published elsewhere. The manuscript is not currently, nor will it be, submitted for publication elsewhere while under consideration by PNTD. All authors have participated in the study and are aware of this submission and will be kept informed of any revisions submitted by the corresponding author. The authors report that there are no conflicts of interest regarding this manuscript.

We look forward to hearing from you regarding these changes.

Sincerely,

Raquel Asunción Lima-Cordón, Ph.D.

University of Vermont

[raqueasu7@gmail.com](mailto:raqueasu7@gmail.com)

**Answers to comments:**

Reviewer's Responses to Questions

Reviewer #1: Suggestions for authors:

*Author summary:*
*Line 54 needs reference. Fix: "Latinamerica" to Latin America*

>>We have changed "Latinamerica" to Latin America

*Line 58: "Wide collection" is a valid point when discussing the countries your specimens were collected in but I think it is better stated as "diverse collection of kissing bugs from United States, Mexico and Central America..." For example, in Mexico alone there are several regional differences that have been described with different populations of triatomines and T. cruzi linkage. Looking at your samples from Mexico, they were collected in the Yucatan but lack the majority of the country. I think it is important that the readers understand this from the beginning.*

>> We have changed "wide collection" to “diverse collection” Now the sentence reads: “Our diverse collection of kissing bugs from North American (United States and Mexico) and Central America …”

Introduction:
*Line 70: even though "10,000 deaths" are often cited for annual mortality, this is likely grossly inaccurate and several experts acknowledge this fact. I think it is important that we recognize this and state this an estimation but mortality is truly unknown.*

>>We have added that 10,000 deaths is an estimate of the mortality. Now the sentence reads: “Trypanosoma cruzi, a hemoflagellated protozoan parasite and the causative agent of Chagas disease, is estimated to be responsible for over 10,000 of these deaths although the actual mortality is unknown [1, 3].”

Line 72: I agree that certain lineages are associated with distinct patterns of clinical disease but this is not always the case. For example we do see gastrointestinal disease in those who were likely infected in regions of TcI predominant regions. I think it is better said that "can be associated with distinct parasite lineages..."

>> We have changed "are" to “can be” Now the sentence read: “… can be associated with distinct parasite lineages …”

Methodology:
Line 139-141: Will need more details pertaining to sample sites. What regions of the U.S., Mexico, and Central American countries (states). Figure 1 needs revamping and is an important visual element of the study. I do not think it is useful as it is being presented. I suggest adding GIS mapping for species collected based off region as it pertains to number sequenced and data from GeneBank. Some regions had more sequencing than others.

>> We have added information regarding the states where samples were collected to the S1 Table when available, however some specimens lack detailed information.

>>We have updated figure 1 focusing on the geographic distribution of field-collected triatomines sampled across North America and Central America, highlighting the states at each country where triatomines were collected.

Why was there a section for "Genetic structure of the Tc I lineage within Central America" and not one for describing North America? Need further clarification.

>> We have we have excluded “within Central America” from the subtitle.

Results:
*Line 155 and Line 345: I am confused with two figure 1. Are we missing a figure? I think authors met Fig 3?
Line 297 and Line 376: I think we have similar issue. Line 376 is Fig 4.
Line 398: Fig 6?*

>> Figures were submitted as individual file and not included in the main manuscript on the initial submission. However, figure captions were inserted in the manuscript text, immediately following the paragraph where the figure was first cited as directed on the submission-guideline document.

Discussion:
Need clearly defined limitations of the study discussed in this section. There are several limitations, which includes sample size. One strength is the data presented from samples collected in Guatemala and El Salvador, but also a limitation as other sampled regions did not have the same variables for analysis.

>> We have included a sentence at the conclusion section addressing the limited sample size for some regions. The sentence reads as follows:

“It would be interesting to test if such genetic population structure is supported when other sampled regions that did not have the same variables for analysis in this study are included.”

I agree with the findings of the author and appreciate that they used the terminology "suggest" and their findings "support" regional evolutionary patterns for T. cruzi lineage. Something not discussed was Mexico and the distinct lineages found in this diverse region that is endemic to Chagas disease. This needs to be considered in the discussion as well as including some data presented by other researchers, such as work done by Herrera and Dumonteil. In the United States, how did your findings correlate with similar investigations looking at phylogeny of T. cruzi strains? This is why I think it is important to include which states those samples were collected in. The U.S. has a very diverse population of triatomines among a large area of geography.

>> We now include data presented by Majeau et al. 2021 from their study “Assessing Trypanosoma cruzi Parasite Diversity through Comparative Genomics: Implications for Disease Epidemiology and Diagnostics”.

Conclusion:
*Line 448: Consistency with the way we describe our Chagas regions. I suggest, "North America, Mexico, and Central America". Triatomines are increasingly being found in regions we did not think they existed. Like certain Caribbean islands and possibly Canada someday with global warming and environmental changes*.

>> We decided to keep North America as United States and Mexico as described in the methodology “Sample sites and *T. cruzi* from insect vectors” section. Samples classified as North America (from United States and Mexico) are based on not only on the collection location of the insect vectors but using biogeographic areas described previously by Morrone (2006). This was stated on lines 246-247. Thus, we consider valid to include Mexico under the North America umbrella based on biogeography.

Line 450 - 454: I agree the findings support the idea of regional diversification and separation of NA and CA from SA lineage. Again, I go back to Mexico, as T. cruzi in Mexico specifically has likely a similar regional diversification pattern. I would also suggest this is likely in the United States as well. This is why I having a hard time putting Mexico under the umbrella of "North America". I would suggest separating these two regions for this paper.

>>We consider that separating Mexico from United States would be ideal for local scale analysis. However, as this reviewer mentioned above, one limitation is the sample size from Mexico. Instead we have added a suggestion at the end of the discussion related to this comment.

Interesting findings are reported with TcBat, which will add to the literature on new region of isolation in Guatemala.

>> Nothing to add to this comment.

General recommendations:

*When discussing North America and Central America, will need to be consistent throughout manuscript in regards to using abbreviations. Also need to be consistent with order at which you are discussing the regions studied. In regards to Mexico, I am not sure how you are going to present this data. In some instances Mexico is considered North America, and in others it is not. When discussing North America with regards to the western hemisphere, it also includes countries in Central America and the Caribbean islands. This can be tricky when reporting data for Chagas disease. My suggestion is be consistent throughout manuscript. North America, Mexico, and Central America is likely the best approach.*

>> We now spell out North America, Central America and South America throughout the manuscript rather than use abbreviations and are consistent with the order in which we discuss the regions.

>>As mentioned above, we have decided to keep North America as United States and Mexico as described in the methodology “Sample sites and *T. cruzi* from insect vectors” section. Samples classified as North America (from United States and Mexico) are based on not only on the collection location of the insect vectors but using biogeographic areas described previously by Morrone (2006). This was stated on lines 246-247. Thus, we consider it valid to include Mexico under the North America umbrella based on biogeography.

*I would also be consistent when describing the vector. I would suggest using triatomine throughout.*

>>We have changed vector to triatomines and keep it consistent throughout the manuscript.

*Please ensure all the names of the authors and those found in the acknowledgements are spelled correctly*.

>>names have been checked to make sure they are spelled correctly.

Reviewer #2:

*Line 22: Be consistent with your abbreviations.*

>> We now spell out North America, Central America and South America in regards to using abbreviations throughout the manuscript as well as being consistent with the order in which we discuss the regions studied.

*Line 174: Where did the authors access reference sequences for TcBat?*

>>As stated at the results section “A phylogeny based on the COII gene only clustered this sample (100% identity) with the only other TcBat COII sequence available, strain Tcc 1994, which was isolated from a bat from Sao Paulo, Brazil (KT337307.1) (S4 Fig).”

*Line 256: Was genomic structure evaluated by triatomines species? If not, would it be possible for authors to run this analysis?*

>> genomic structure was not evaluated by triatomines species because of the limited triatomine species sampling from all the locations.

*Line 325: That’s an interesting finding! Makes me curious if it’s acute, oral outbreak associated vector in both locations-??*

>> No comments to add.

*Line 370: Country names should be included*

>> We have included the location names Jutiapa, Chiquimula, Santa Ana and Sonsonate
